# Variation in Genetic Mechanisms for Plumage Polymorphism in Skuas (*Stercorarius*)

**DOI:** 10.1093/jhered/esab038

**Published:** 2021-08-03

**Authors:** Kirstin Janssen, Jan Ove Bustnes, Nicholas I Mundy

**Affiliations:** 1 Department of Natural Sciences, Tromsø University Museum, NO-9037 Tromsø, Norway; 2 Centre of Forensic Genetics, Institute of Medical Biology, Faculty of Health Sciences, UIT The Arctic University of Norway, NO-9037 Tromsø, Norway; 3 Norwegian Institute for Nature Research, The Fram Centre, NO-9296 Tromsø,Norway; 4 Department of Zoology, University of Cambridge, Cambridge, UK

**Keywords:** *MC1R*, melanin, repeatability of evolution, skua, *TYRP1*

## Abstract

Coloration is evolutionarily labile and so provides an excellent trait for examining the repeatability of evolution. Here, we investigate the repeatability of the evolution of polymorphic variation in ventral plumage coloration in skuas (*Stercorarius*: Stercorariidae). In 2 species, arctic (*S. parasiticus*) and pomarine skuas (*S. pomarinus*), plumage polymorphism was previously shown to be associated with coding changes at the melanocortin-1 receptor (*MC1R*) locus. Here, we show that polymorphism in a third species, the south polar skua (*S. maccormicki*), is not associated with coding variation at *MC1R* or with variation at a *Z*-linked second candidate locus, tyrosinase-related protein 1 (*TYRP1*). Hence, convergent evolution of plumage polymorphisms in skuas is only partly repeatable at the level of the genetic locus involved. Interestingly, the pattern of repeatability in skuas is aligned not with phylogeny but with the nature of the phenotypic variation. In particular, south polar skuas show a strong sex bias to coloration that is absent in the other species, and it may be that this has a unique genetic architecture.

In recent years, great strides have been made in establishing the genetic basis of color polymorphisms in wild populations in a wide variety of species (Hubbard et al. 2010; San-Jose and Roulin 2017), making coloration an important model system for understanding the genetic basis of adaptation. An important unresolved issue is the repeatability of color evolution, that is, the extent to which convergent changes in phenotype are due to convergent genetic changes (Stern and Orgogozo 2009; Arnaud and Orgogozo 2013). There are cases where color evolution is at least to some extent repeatable, particularly at the level of the loci involved (Manceau et al. 2010; Kronforst et al. 2012). However, far more evidence is needed to quantify this issue and, more importantly, to explain the causes of the patterns that are found. For terrestrial vertebrates, the majority of information on the genetic basis of color variation comes from melanin-based variation, although progress is beginning to be made for other coloration mechanisms (e.g., carotenoids; Twyman et al. 2018).

Shared color polymorphisms among related species are a special case in relation to repeatability. In such cases, there may have been convergent evolution of the polymorphism, but an alternative possibility is that the polymorphism arose a single time in a common ancestor, and was subsequently maintained across speciation events (Jamie and Meier 2020). While such “trans-specific” evolution is well-known for some highly polymorphic systems (e.g., MHC, Klein et al. 2007; self-incompatibility, Richman et al. 1996), the data for color polymorphisms are currently limited.

Birds are an excellent group of vertebrates to address these issues since there are several avian genera with a similar polymorphic color variation that is shared across multiple related species (e.g., buzzards [*Buteo*], sparrowhawks [*Accipiter*], owls [*Tyto*], and egrets [*Aigretta*]; Roulin 2004). Genera with multiple polymorphic species, for which there are some genetic data, include skuas (*Stercorarius*; Mundy et al. 2004; Janssen and Mundy 2017), falcons (*Falco*; Gangoso et al. 2011; Johnson et al. 2012; Zhan et al. 2012), and boobies (*Sula*; Baião et al. 2007;Baião and Parker 2012). There are also examples of convergent evolution in different populations of the same species (e.g., Uy et al. 2016). Results from these studies are variable, some implicating the same locus in convergent evolution, whereas in others different loci are involved.

The skuas (*Stercorarius*) are kleptoparasitic seabirds that include 3 species with well-defined ventral melanin-based plumage polymorphisms (Furness 1987). It has previously been shown that in 2 polymorphic species, the arctic (*S. parasiticus*) and pomarine skua (*S. pomarinus*), color variation has evolved independently by mutations in the melanocortin-1 receptor (*MC1R*), and some of the mutations implicated in color variation have occurred independently in the 2 lineages (Mundy et al. 2004; Janssen and Mundy 2013, 2017). The third species is the south polar skua (*S. maccormicki*), one of the great skua species (previously comprising the genus *Catharacta*), which is more closely related to pomarine than arctic skuas (Braun and Brumfield 1998). Three color morphs have been defined in the south polar skua, with ventral plumage coloration varying from pale buff in the pale morph to dark brown in the dark morph (Figure 1; Ainley et al. 1985; Olsen and Larsson 1997). As in the arctic skua, there is a latitudinal cline in plumage coloration, with dark morph birds more common in warmer areas and pale morph birds more common in cooler areas. In addition, and unlike the arctic and pomarine skuas, there is sex-specific variation in plumage color in south polar skuas, with males being on average darker than females (although the 3 color morphs are present in both sexes, Ainley et al. 1985). While plumage coloration in south polar skuas is presumed to be under genetic control, there are no studies of genetic transmission of coloration across generations. Additionally, there is little information on the fitness consequences of plumage color variation in this species. In the best-studied species, the arctic skua, the most favored adaptive hypothesis for the maintenance of the polymorphism is apostatic selection (Árnason 1978, Arcos 2007), a type of negative frequency-dependent selection in which rare morphs would have an advantage in pursuit of prey, but there is also some evidence for sex-specific selection on coloration (Janssen et al. 2006). We previously showed that the plumage polymorphisms in the arctic and pomarine skua were not present in their common ancestor (Janssen and Mundy 2017), but it remains possible that a polymorphism arose in the ancestor of the pomarine and great skuas and was retained in the south polar skua lineage.

**Figure 1.**
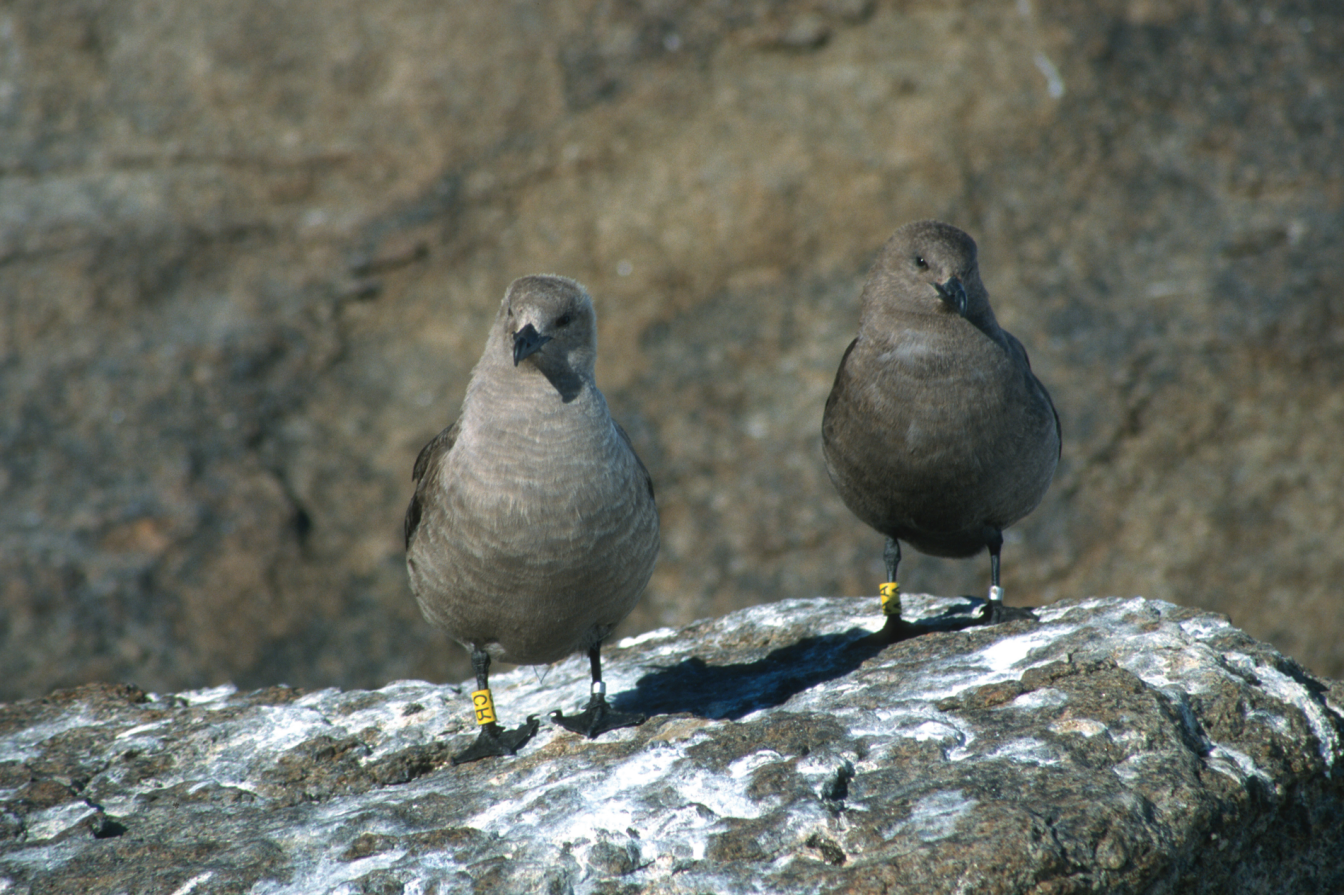
Typical color variation in the south polar skua. Pale morph female on the left and dark morph male on the right. (*Photo credit:* J.O.B.).

The genetic basis of plumage color variation in south polar skuas has not been previously investigated. Here, we examine whether the evolution of plumage polymorphism in this species has occurred by the same genetic mechanisms as in the other skuas. In order to achieve this, we assay variation at 2 candidate coloration loci. The first candidate, *MC1R*, was previously implicated in color variation in arctic and pomarine skuas and has been shown to have sex-dependent effects on coloration in barn owls (*Tyto alba*; San-Jose et al. 2015). The second candidate, tyrosinase-related protein 1 (*TYRP1*), was chosen since it has been associated with melanin-based color variation in other birds (Buggiotti 2007; Nadeau et al. 2007b; Delmore et al. 2016), and, since it is *Z*-linked, it is a good candidate for sex-related variation in coloration. MC1R has a critical role in regulating the type of pigment synthesized by melanocytes, whereas TYRP1 is one of the enzymes required for eumelanin synthesis (Hubbard et al. 2010).

## Materials and Methods

### Samples

Plumage phenotype data and blood samples from adult south polar skuas caught on the nest were collected at Svarthamaren 71°53′0″S, 5°10′0″E in Dronning Maud Land, Antarctica, in December 2001 and January 2002 (Bustnes et al. 2006). The plumage phenotype of 64 birds was visually categorized as “pale,” “intermediate,” or “dark” by a single experienced observer (J.O.B.). In south polar skuas, dark morph individuals are distinct, whereas continuous variation is present between pale and intermediate morphs (Ainley et al. 1985). The individuals’ sex was determined by molecular analyses as described previously (Bustnes et al. 2006). A total of 25 individuals comprising 8 pale birds (5 females and 3 males), 7 intermediate birds (5 females and 2 males), and 10 dark birds (2 females and 8 males) were chosen for the analysis of candidate pigmentation genes.

### Laboratory and Statistical Methods

Genomic DNA was extracted from blood samples using standard sodium dodecyl sulfate (SDS) lysis and proteinase K digestion followed by phenol–chloroform extraction and ethanol precipitation protocols (Sambrook et al. 1989). *MC1R* genotypes from the 25 individuals were obtained as part of a previous study (Janssen and Mundy 2017). Briefly, 846 bp of the coding sequence of *MC1R* gene including all of the sites known to be involved in color variation in other vertebrates, and 285 bp of 5′ upstream sequence was amplified and directly sequenced.

For *TYRP1*, exon 3 was initially sequenced in the 25 individuals since this exon is the site of coloration-changing mutations in other birds and mammals (Gratten et al. 2007; Nadeau et al. 2007b). Subsequent analyses extended to exons 2, 4, 5, and 6, screening one individual of each sex-plumage phenotype combination for each exon (*N* = 6). Primers used to amplify and sequence exons 2 to 6 are listed in Table 1. Amplifications were carried out in 15 μL reaction volumes containing 1× CoralLoad PCR, including 1.5 mM MgCl_2_, 1× Q-solution, 200 μM of each dNTP, 0.4 μM of each primer, 3.75 units Taq (Qiagen Taq PCR Core kit) (Qiagen, Manchester, UK), and approximately 50 ng DNA. For detailed Polymerase chain reaction (PCR) conditions, see Table 1. Excess primers and nucleotides were either removed using the QIAGEN purification kit or EXOSAP-IT (Amersham Biosciences, Little Chalfont, UK). All amplification products were Sanger sequenced on both strands with Big Dye v.3.1 (Applied Biosystems, Warrington, UK) at the sequencing facilities of the University of Oxford, UK, and the University Hospital of Tromsø, Norway.

**Table 1.** *TYRP1* PCR and sequencing primers

Primer name	Primer sequence (5′-3′)	bp	Position	Amplifying	Designed from	PCR conditions^*a*^
TYR1332F^*b*^	GAATGGAACAGGAGGGCAAAC	21	Exon 5	Intron V	Chicken messenger RNA (mRNA)	T_A_ = 61 °C for 30 s; T_E_ = 72 °C for 90 s
TYR1332R^*b*^	TCCAATAGGGGCATTCTCCAG	21	Exon 6	Intron V	Chicken mRNA	
**TYR600F** ^*b*^	GAAGGGCAGAAGGAAGGAAC	20	Intron V		Skua DNA	
**TYR1070F** ^*b*^	TTCCACTGGTATTCATATCAGCTAC	25	Intron V		Skua DNA	
**TYR740R** ^*b*^	TGCACAGACATAAGGGTTACACAG	24	Intron V		Skua DNA	
**TYR1260R** ^*b*^	TGGTGTGACAGATACAGAATGG	22	Intron V		Skua DNA	
TYRe1F	GCTTCTTCAACCAAACCTG	19	Exon 1	Intron I	Chicken & zebra finch mRNA	Touch down, T_A_ = 58 °C for 30s; T_E_ = 72 °C for 180 s
TYRe2R	TAATAATGAGACCACACAAAGTAG	24	Exon 2	Intron I	Chicken & zebra finch mRNA	
TYRe2F	AAGGAGACTTTTTGTGAATGC	21	Exon 2	Intron II	Chicken & zebra finch mRNA	T_A_ = 60 °C for 30 s; T_E_ = 72 °C for 90 s
TYRe3R	CGCCACTGAGAGAAGATTG	19	Exon 3	Intron II	Chicken & zebra finch mRNA	
TYRe3F	CAATCTTCTCTCAGTGGCG	19	Exon 3	Intron III	Chicken & zebra finch mRNA	T_A_ = 55 °C for 30 s, T_E_ = 72 °C for 90 s
TYRi4R(skua)	GACTACAAACTCATACTTCCGAC	23	Exon 4	Intron III	Skua DNA	
TYRe4F	TCTATTCCAATTCAACAGACAGTTT	25	Exon 4	Intron IV	Chicken & zebra finch mRNA	T_A_ = 58 °C for 30 s; T_E_ = 72 °C for 120 s no Q-solution
TYRi5skuaR	ATACCTTCTCAGCCACTCATG	21	Intron V	Intron IV	Chicken & zebra finch mRNA	
TYRi5skuaF	AGAAAGGTTTACAATCTACTGGTG	24	Intron V	Exon 6	Chicken & zebra finch mRNA	T_A_ = 56 °C for 30 s; T_E_ = 72 °C for 60 s
TYRe7	ATGCAGCAGCAGCAAAGATA	20	Exon 7	Exon 6	Chicken & zebra finch mRNA	
TYRie2F	GCTTTATTTTTGTTTGGCTTA	21	Intron I	Exon 2	Skua DNA	T_A_ = 61 °C for 30 s; T_E_ = 72 °C for 90 s
TYRie2R	TGCTATCATTTTTATGTATTGGAA	24	Intron II	Exon 2	Skua DNA	
TYRie3F	GTATCCCTTTTCCCTTACTTTT	22	Intron II	Exon 3	Skua DNA	T_A_ = 50 °C for 30 s; T_E_ = 72 °C for 60 s
TYRie3R	ATTTTGAACTTCTTGGTGCC	20	Intron III	Exon 3	Skua DNA	
TYRie4F	AGTATTGTTTTCGCTCTTCTCTTC	24	Intron III	Exon 4	Skua DNA	T_A_ = 50 °C for 30 s; T_E_ = 72 °C for 60 s
TYRie4R	TTGTTCCAGATGGTTTTATTTG	22	Intron IV	Exon 4	Skua DNA	
TYRie5F	ATCCCAGCAGCCTTGCACTC	20	Intron IV	Exon 5	Skua DNA	Touch down, T_A_ = 58 °C for 30 s, T_E_ = 72 °C for 60 s
TYRie5R	CTTCCACGGTTACACAATCTTT	22	Intron V	Exon 5	Skua DNA	
TYRie6F	ATTTTGATTTCAGTTACAGAAGTGT	25	Intron V	Exon 6	Skua DNA	Touch down, T_A_ = 58 °C for 30 s; T_E_ = 72 °C for 60 s
TYRie6R	ATTGAAGTGGATAGTGGGAGC	21	Intron VI	Exon 6	Skua DNA	

Primers used for sequencing only are shown in bold.

^*a*^PCR conditions: 3 min of initial denaturation at 94 °C, 40 cycles consisting of 94 °C for 30 s, annealing (T_A_) and extension (T_E_), followed by 5-min final extension.

^*b*^ Janssen and Mundy (2017).

Sequence data were edited with Bioedit v7.2.5 (Hall 1999). Tests for association between plumage coloration and sex, and between plumage coloration and genotype, were conducted using Fisher’s exact tests with the fisher.test function in R studio (RStudio Team 2020).

## Results

### Association Between Sex and Plumage Coloration

In the full sample of 64 individuals, there were 15 dark, 3 intermediate, and 4 pale males and 2 dark, 6 intermediate, and 34 pale females. Coloration was significantly associated with sex, with males being darker than females (2 × 3 Fisher’s exact test, *P* < 0.001), which concurs with previous findings (Ainley et al. 1985).

### Association Between Genetic Variation at Candidate Loci and Plumage Coloration

At the *MC1R* locus (1171 bp, *N* = 25), there were 2 variable sites in south polar skuas, one non-synonymous Single-nucleotide polymorphism (SNP), leading to a glutamate to lysine (E12K) substitution and one synonymous SNP, defining 2 haplotypes at *MC1R*. One haplotype (with E12; Genbank Accession MG515660.1) was sequenced once from a single individual (a dark morph male) and is shared with the closely related monomorphic brown skua (*S. lonnbergii*; Janssen and Mundy 2017). The second haplotype (with K12; Genbank Accession MG515661.1) was present as 49 copies in our sample and has not been found in other skua species. Thus, unlike the situation with arctic and pomarine skuas, *MC1R* variation is not associated with plumage color variation in the south polar skua.

The 5 exons at *TYRP1* (exons 2–6) were completely sequenced (exon 3 in *N* = 25 birds; exons 2, 4, 5, and 6 in *N* = 6 birds, total length = 1024 bp). No variable sites were found. Hence, no evidence was found for an association between *TYRP1* and plumage coloration in the south polar skua.

## Discussion

We investigated whether convergent evolution of melanin-based plumage coloration in south polar skuas was attributable to the same locus that accounts for convergent evolution of a similar phenotype in 2 other species of skua. The results clearly indicate that this is not the case and do not implicate *MC1R* as a major effect locus for color variation in south polar skuas. In a sample containing extremes of color variation in both males and females, almost all birds are homozygous for the same *MC1R* allele. One caveat to these findings is that we did not sequence the last 20 codons of *MC1R* (out of a total of 315). However, the gene portion sequenced here contains all of the sites previously shown to be functionally important in birds and other vertebrates (Theron et al. 2001; Mundy 2005; Cibois et al. 2011), so it is unlikely that functional variation is present in this region. In addition, we only sequenced 285 bp of 5′ upstream noncoding sequence of *MC1R*, so it remains possible that *cis*-regulatory mutations elsewhere at the locus affecting *MC1R* expression contribute to the color variation. There is currently little information on *cis*-regulatory mutations at *MC1R* influencing coloration, although some recent whole-genome studies suggest that they may occur in birds (Funk and Taylor 2019). Interestingly, the one divergent allele sequenced (from a dark male) is shared with the brown skua (*S. lonnbergii*), in which this is the only *MC1R* allele reported (Janssen and Mundy 2017). This may be due to introgression since hybridization occurs between south polar and brown skuas (Ritz et al. 2006), although there are other potential mechanisms such as incomplete lineage sorting. The allele has an E12K mutation associated with dark coloration in arctic and pomarine skuas, and brown skuas have uniform dark brown coloration. Although this mutation might plausibly affect coloration in south polar skuas, it is clearly not implicated in the main coloration variation present in this species. Since we have excluded a role for the same coding variants of *MC1R* to be involved in plumage polymorphism in south polar and pomarine skuas, we conclude that the plumage polymorphisms evolved independently in the 2 species.

The color variation in south polar skuas differs in important respects from that in arctic and pomarine skuas. Most importantly, coloration in south polar skuas is sex-biased, which we confirmed here. Although most cases in which *MC1R* variation has been associated with plumage polymorphism do not involve sex differences in coloration, *MC1R* has been occasionally linked to sexual variation in coloration (barn owl [*Tyto alba*]; San-Jose et al. 2015) and so was a plausible candidate for south polar skuas. In addition, *MC1R* has been implicated in other sex-specific effects, such as the evolution of sexual dichromatism in birds (Nadeau et al. 2007a), and sex-specific pain reception in mammals (Mogil et al. 2003). Nevertheless, it is possible that *MC1R* is less likely to be involved in cases of sex-biased coloration. In addition, whereas continuous variation between pale and intermediate morphs is present in south polar skuas, with dark morphs being distinct, in arctic skuas, it is the pale morph that is distinct, and there is continuous variation between intermediate and dark morphs. However, the consequences of this difference for genetic architecture are unclear. In addition to well-known association with discrete color polymorphisms, we note that *MC1R* is a major effect locus underlying quantitative color variation in numerous cases (e.g., dark/intermediate arctic skuas, blue phase snow geese [*Chen caerulescens*], Mundy et al. 2004; beach mice [*Peromyscus polionotus*], Steiner et al. 2007).

The sex-biased coloration informed our choice of another candidate locus, the *TYRP1* locus, which is *Z*-linked in birds (April et al. 1998). We found no variation in the 5 exons of *TYRP1* that were targeted, and hence no evidence that sequence variation in *TYRP1* is associated with the polymorphism. We chose this segment of the locus since it is the site of mutations that lead to color variation in other species, notably Japanese quail (Nadeau et al. 2007b), but cannot rule out the possibility that variation in the remaining exons contributes to color variation. In addition, it remains possible that gene dosage effects on the expression of *TYRP1* among females and males could contribute to sex-specific differences in coloration, which would be interesting to explore further.

An obvious next step to identify genetic variation contributing to the plumage polymorphism in south polar skuas would be to conduct a Genome-wide association study (GWAS), an approach that has succeeded in uncovering candidate loci affecting melanin coloration in other birds (crows, Poelstra et al. 2014; wood warblers, Toews et al. 2016; Swainson’s thrushes, Delmore et al. 2016; and ruff, Küpper et al. 2016). The low level of genetic variation in south polar skuas, which is presumably related to a low effective population size resulting from a founder event (Ritz et al. 2008), would increase the power of this approach (Braun and Brumfield 1998; Janssen and Mundy 2017).

Other studies on polymorphisms within or between multiple avian species offer interesting contrasts. In falcons and boobies, there is evidence that the same locus (*MC1R*) is involved in melanic polymorphisms in multiple species (Baião et al. 2007, 2012; Gangoso et al. 2011; Johnson et al. 2012; Zhan et al. 2012). However, we are not aware of any cases where polymorphism due to the same locus evolved ancestrally and was retained through subsequent speciation(s). In Solomon Islands flycatchers and crows, subspecies or populations in a species complex show evidence of differing loci in the same pathway being involved in convergent evolution of melanic coloration (Uy et al. 2009, 2016; Poelstra et al. 2014; Vijay et al. 2016). All of these examples involve sexually monomorphic coloration. In conclusion, it may be that genetic architecture for sexually varying polymorphic coloration, as in the south polar skua, differs from that for sexually monomorphic coloration.

## Data Availability

We have deposited the new primary data underlying these analyses as follows: DNA sequences: Genbank accession MZ488491.
